# Functional assays to determine the significance of two common *XPC *3'UTR variants found in bladder cancer patients

**DOI:** 10.1186/1471-2350-12-84

**Published:** 2011-06-20

**Authors:** Boling Qiao, Gina B Scott, Faye Elliott, Laurence Vaslin, Johanne Bentley, Janet Hall, D Timothy Bishop, Margaret A Knowles, Anne E Kiltie

**Affiliations:** 1 Section of Experimental Oncology, Leeds Institute of Molecular Medicine, Leeds LS9 7TF, UK; 2 Section of Experimental Oncology, Leeds Institute of Molecular Medicine, Leeds LS9 7TF, UK; 3 Section of Epidemiology and Biostatistics, Leeds Institute of Molecular Medicine, Leeds LS9 7TF, UK; 4 INSERM U612, Centre Universitaire, Orsay 91405, France and Institut Curie, Centre Universitaire, Orsay 91405, France; 5 Section of Experimental Oncology, Leeds Institute of Molecular Medicine, Leeds LS9 7TF, UK; 6 INSERM U612, Centre Universitaire, Orsay 91405, France and Institut Curie, Centre Universitaire, Orsay 91405, France; 7 Section of Epidemiology and Biostatistics, Leeds Institute of Molecular Medicine, Leeds LS9 7TF, UK; 8 Section of Experimental Oncology, Leeds Institute of Molecular Medicine, Leeds LS9 7TF, UK; 9 Section of Experimental Oncology, Leeds Institute of Molecular Medicine, Leeds LS9 7TF, United Kingdom and Gray Institute for Radiation Oncology and Biology, Department of Oncology, University of Oxford OX3 7DQ, UK

## Abstract

**Background:**

*XPC *is involved in the nucleotide excision repair of DNA damaged by carcinogens known to cause bladder cancer. Individuals homozygous for the variant allele of *XPC *c.1496C > T (p.Ala499Val) were shown in a large pooled analysis to have an increased bladder cancer risk, and we found two 3'UTR variants, *611T > A and c.*618A > G, to be in strong linkage disequilibrium with c.1496T. Here we determined if these two 3'UTR variants can affect mRNA stability and assessed the impact of all three variants on mRNA and protein expression.

**Methods:**

*In vitro *mRNA stability assays were performed and mRNA and protein expression measured both in plasmid-based assays and in lymphocytes and lymphoblastoid cell lines from bladder and breast cancer patients.

**Results:**

The two 3'UTR variants were associated with reduced protein and mRNA expression in plasmid-based assays, suggesting an effect on mRNA stability and/or transcription/translation. A near-significant reduction in XPC protein expression (p = 0.058) was detected in lymphoblastoid cell lines homozygous for these alleles but no differences in mRNA stability in these lines was found or in mRNA or protein levels in lymphocytes heterozygous for these alleles.

**Conclusion:**

The two 3'UTR variants may be the variants underlying the association of c.1496C > T and bladder cancer risk acting via a mechanism modulating protein expression.

## Background

Transitional cell carcinoma of the bladder is the fourth commonest cancer in men in the United Kingdom (http://info.cancerresearchuk.org/cancerstats/types/bladder/index.htm) with cigarette smoking and occupational chemical exposure being major risk factors. The metabolism of such carcinogens generates many bulky DNA adducts which are repaired by the nucleotide excision repair (NER) pathway [1]. A key NER protein, XPC, recognizes and binds to helix-distorting DNA adducts [2] and is involved in repair of oxidative DNA damage formed following carcinogen exposure [3].

We previously studied 23 *XPC *SNPs in 547 bladder cancer cases and 579 controls, and found that homozygous carriage of the variant alleles of c.1496C > T (p.Ala499Val, rs2228000) and two 3'-untranslated region (UTR) polymorphisms, c.*611T > A (rs2470352) and c.*618A > G (rs2470458; previously named Ex15-184 and Ex15-177 respectively), was associated with increased bladder cancer risk [4]. Recently the effect of the c.1496T variant has been confirmed in a large pooled analysis [5]. However, this variant is not predicted to have functional effects by a number of analytical tools, and in support of this, we recently demonstrated that the c.1496 T allele had no influence on recruitment of GFP-tagged XPC to sites of focal 408 nm laser damage in a cell-based assay [6]. We therefore wished to determine whether the two 3'UTR variants in strong linkage disequilibrium with c.1496T had an impact on mRNA stability and mRNA and protein expression, thus potentially being the variants underlying the association between c.1496T and increased bladder cancer risk.

## Methods

### Cell lines

Cells were grown at 37°C in a 5% CO_2 _humidified atmosphere. Lymphoblastoid cell lines (LCLs) established from breast cancer patients [7] were cultured in RPMI 1640, 15% heat inactivated fetal bovine serum (FBS), 1% L-glutamine + penicillin/streptomycin. GM15983 SV40-transformed XP-C cells (2 bp frameshift at codon 431, purchased from the Coriell Institute, NJ) [8], were cultured in Dulbecco's Modified Eagle's Medium (Sigma), 10% FBS and 1% L-glutamine. Daudi human lymphoblastoid cells, purchased from ATCC, and RT112M bladder cancer cells were cultured in RPMI 1640, 10% FBS, 1% L-glutamine.

### 3'UTR plasmid reporter system and FACS analysis

The plasmid reporter system and analysis has been described in detail [6]. Briefly, the 5'- and 3'UTR regions of XPC were cloned into plasmid pTH-GFPa and the changes c.*611T > A and c.*618A > G introduced by site-directed mutagenesis. Plasmids were transfected into RT112 bladder cancer cells, using Fugene transfection reagent and cells analysed by FACS for mean fluorescent intensity (MFI) after overnight incubation. RNA was isolated from parallel cultures and used to synthesise cDNA for quantitative real-time RT-PCR with SYBR green as the fluorescent reporter, to determine the Ct value, and *GFP *mRNA quantified relative to the housekeeping gene *36B4*.

### XPC mRNA stability assays

BCL and GM15983 cells were plated into 6-well tissue culture plates and 24-hours later treated with actinomycin D (ActD, 1 μg/ml) (Sigma, UK). Cells were harvested at 0 (control, untreated), 2, 4, 6 and 8 hours later and total RNA was extracted using a PerfectPure RNA Cultured Cell Kit (Flowgen Bioscience, Nottingham, UK) and used to synthesize cDNA using Superscript II (Invitrogen, UK). *XPC *mRNA was quantified using quantitative real-time RT-PCR (Table 1), with *XPC *cDNA levels normalized to *SDHA*.

**Table 1:** Primers for real-time RT-PCR

Primer	Assay	Primer Sequences	Direction
XPC-F	XPC	5'-TACTCCCATCCCGTGACT-3'	Forward
XPC-R	XPC	5'-GAGCCCGCTTCTCCTTT-3'	Reverse
SDHA-F	SDHA	5'-TGGGAACAAGAGGGCATCTG-3'	Forward
SDHA-R	SDHA	5'-CCACCACTGCATCAAATTCATG-3'	Reverse
GFP-F	GFP	5'-CAACCACTACCTGAGCACCCAGTC-3'	Forward
GFP-R	GFP	5'-GGCGGCGGTCAGGAACTC-3'	Reverse
36B4-F	36B4	5'-GAAACTCTGCATTCTCGCTTCC-3'	Forward
36B4-R	36B4	5'-GATGCAACAGTTGGGTAGCCA-3'	Reverse

### Patient sample collection and processing

Local ethical approval was granted by the Leeds Teaching Hospitals Local Research Ethics Committee (LREC) and informed consent obtained. A 20 ml blood sample was collected from 49 patients with a previous history of bladder cancer.

### DNA preparation and sequencing

Genomic DNA was isolated from 200 μl whole blood from bladder cancer patients using a QIAamp DNA Micro Kit (Qiagen). PCR reactions were carried out in a total volume of 20 μl containing 20 ng of DNA, 10 μl HotstarTaq Master Mix (Qiagen), and 0.5 μM forward and reverse primers (Table 2). Thermal cycling parameters were 15 min at 95°C, followed by 45 cycles of 95°C for 30 seconds, optimal primer annealing temperature for 30 seconds and 72°C for 30 seconds, followed by a final extension of 72°C for 10 minutes. The PCR products were Sanger sequenced using ABI BigDye Terminator (Applied Biosystems, USA) and compared to the reference sequence (GenBank accession number AC090645).

**Table 2:** *XPC *primers, amplicon size and annealing temperatures

SNPs	Forward primer sequence	Reverse primer sequence	Amplicon size (bp)	Annealing temp (°C)
c.1496C > T	GACAAGCAGGAGAAGGCAAC	ACCATCGCTGCACATTTTCT	333	63
c.*611T > A c.*618A > G	AATGCGCTGATCGTTTCTT	AGAGCCAAATCTTTAGATAAATGC	420	61

Primer pairs designed based on the *XPC *sequence (GenBank accession number AC090645).

### RNA extraction and quantitative real-time RT-PCR

Peripheral blood mononuclear cells (PBMC) from bladder cancer patients were isolated from 19.8 ml whole peripheral blood using Lymphoprep™ tubes (Greiner Bio-one Ltd, Brunel Way, UK) with Ficoll gradient centrifugation. The cells were then incubated at 37°C/5% CO_2 _in RPMI 1640, 10% heat-inactivated FBS, 1% phytohemagglutinin (Sigma) for 72 hours. Total RNA was extracted using the PerfectPure RNA Blood Kit (Flowgen Bioscience, Nottingham, UK) and used to synthesize cDNA using Superscript II (Invitrogen, UK). For the LCLs, total RNA was extracted similarly, after cells were grown in RPMI 1640 with 10% heat-inactivated FBS, at 37°C/5% CO_2 _for 3-5 days.

mRNA levels were quantified using gene-specific primer pairs by real-time RT-PCR (Table 1) on a 7500 Real Time PCR System using SYBR Green I (Applied Biosystems, CA). PCR reactions were performed using 5 μl cDNA (corresponding to 50 ng RNA), 300 pM primers and SYBR Green matrix (AB Applied Biosystems, CA) per reaction in triplicates of 25 μl volume, following the manufacturer's protocol. Thermal cycling parameters were 50°C for 2 min, denaturation at 95°C for 10 min, then 40 cycles of amplification (95°C for 15 seconds and annealing/extension at 60°C for 1 min). Results were analyzed using the comparative *Ct *method after validation, with *XPC *cDNA levels normalized to those of *SDHA*.

### Protein extraction and western blotting

The LCLs and PHA-stimulated PBMC from bladder cancer patients were lysed in RIPA buffer, containing 1% protease and phosphatase inhibitors (Sigma, UK), the lysate clarified by centrifugation and stored at -80°C. Samples of 50 μg protein were heated to 95°C for 5 minutes, resolved on 7.5% or 10% (w/v) polyacrylamide Tris gels and transferred onto Hybond-C membrane (Amersham, Little Chalfont, UK) at 100 V for 1 h in ice-cold transfer buffer (25 mM Tris, 192 mM glycine). Following blocking in Odyssey blocking buffer (Li-cor Biosciences, Cambridge, UK) for 1 h, blots were incubated for 1 h in antibody buffer [1:1 v/v Odyssey blocking buffer: phosphate buffered saline, 0.1% v/v Tween-20 (PBS-T)] containing primary antibody: XPC 1:1,000 (rabbit polyclonal C-terminal anti-XPC, Sigma, UK) or β-actin 1:10,000 (mouse monoclonal clone AC-15, Sigma, UK). After three 20 min washes in PBS-T, blots were incubated with secondary antibody (1:5,000; 680-conjugated goat anti-rabbit IgG (Invitrogen, Paisley, UK) or IRDye 800-conjugated rabbit anti-mouse IgG (Rockland, Peterborough, UK)). Following three washes in PBS-T, XPC protein was detected and quantified using the Odyssey infra-red detection system (Li-cor Biosciences UK Ltd, Cambridge, UK) and bands normalized to β-actin levels.

### Statistical analysis

All statistical analyses were performed using Excel and SPSS software. Student's t-test was used for comparison of means. Pair-wise correlations between the relative expressions levels were analyzed by the Pearson correlation coefficient. P values < 0.05 were considered statistically significant.

## Results

**Figure 1:**
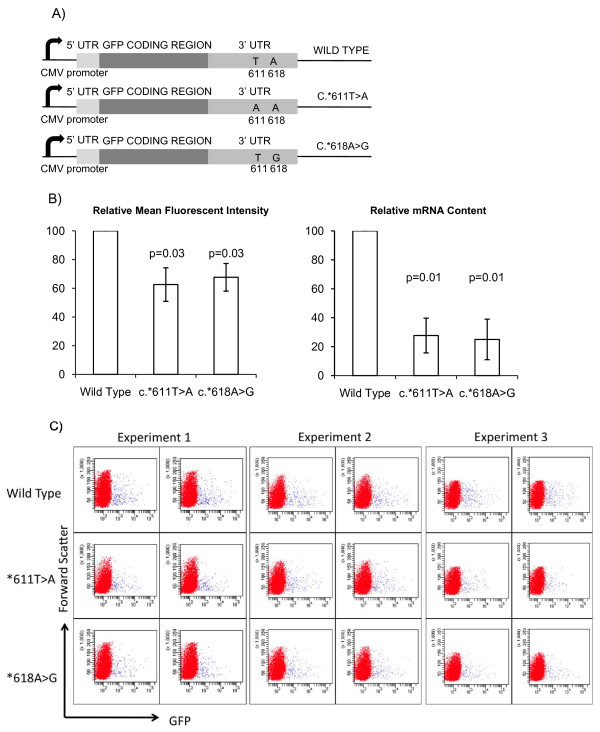
**3'UTR plasmid reporter assay**. A) Schematic diagram of pTH-GFPa constructs used in mRNA translational efficiency assays. B) 3'UTR plasmid reporter system and FACS analysis (n = 3, duplicate wells analysed in each experiment, 2-tailed t-test, error bars SD); (i) Mean fluorescence intensity (MFI) of RT112 cells transfected with *GFP*-flanked by mutant 3'UTR sequences relative to that of *GFP*-flanked by wild-type XPC UTR sequences; (ii) Relative levels of *GFP *mRNA to *36B4 *mRNA analysed by quantitative RT-PCR using the ΔΔCt method. C) Flow cytometry data of individual transfections (n = 3, duplicate wells analysed in each experiment). GFP fluorescence measured on × axis (forward scatter on y axis). See Table 3 for mean fluorescent intensity values.

We used a plasmid based assay, in which the individual 3'UTR variants (Figure 1A) were transfected into RT112 bladder cancer cells to assess their impact on the relative protein expression assayed by FACS and relative mRNA content assayed by quantitative real-time RT-PCR (Figure 1B, C and Table 3. There were significant reductions in both protein (p = 0.03) and mRNA expression (p = 0.01) in the presence of either 3'UTR SNP variant compared to those of wild-type 3'UTR.

**Table 3:** Mean Fluorescent Intensity (MFI) of GFP transfected cells

	Expt1	Mean MFI	%	Expt2	Mean MFI	%	Expt3	Mean MFI	%	Mean % (± StDev)
Wild Type	3577	4145.5	100	5736	6165.5	100	3953	4135.5	100	100

	4706			6595			4334			

*611T > A	2541	2735	66.04	4196	4469	72.48	2168	2059	49.69	62.74 (± 11.75)

	2929			4742			1950			

*618A > G	3681	3066	74.03	3345	3832	62.15	3077	3265	78.8	71.66 (± 8.57)

	2451			4319			3453			

Duplicate wells were analysed in each experiment and the Wild Type MFI used as the maximum value of 100%.

**Figure 2:**
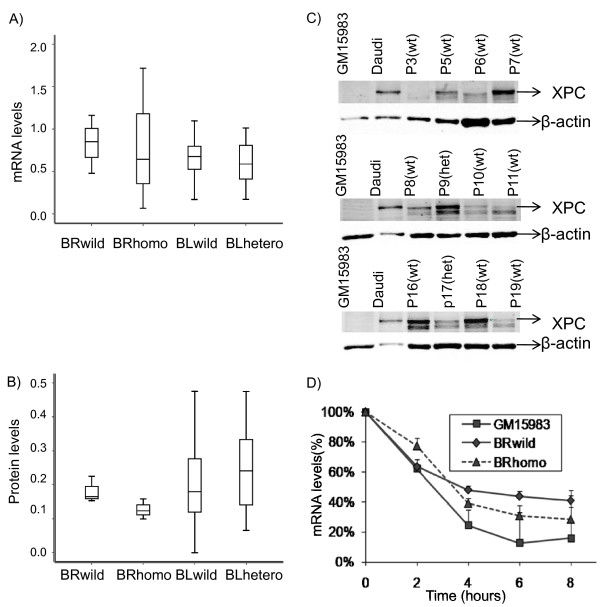
**Breast cancer patient lymphoblastoid cell lines and bladder cancer patient clinical samples**. Comparison of: A) mRNA levels, by real time RT-PCR (n = 3), and B) XPC protein levels, by western blotting (n = 3), in LCLs from breast cancer (BR) patients and PBMC from bladder cancer (BL) patients, with wild-type, heterozygous or homologous alleles of *XPC *c.1496C > T and the two 3'UTR polymorphisms (c.*611T > A and c.*618A > G). Mean mRNA levels were normalized to *SDHA *and compared to that of Daudi cells. Mean protein levels were normalized to β-actin and compared to that of Daudi cells. Thick line represents median, box represents interquartile range and errors bars represent 95% confidence intervals. C) XPC assessment by western blotting in PBMC from some of the bladder cancer patients, with wild-type (wt) and heterozygous (het) alleles of XPC c.1496C > T and the two 3'UTR polymorphisms (c.*611T > A and c.*618A > G). Top band only was quantified. GM15983 cells were used as negative controls and Daudi cells were used as positive controls. D) mRNA stability assays in LCLs from breast cancer patients and GM15983 XP-C cells. *XPC *mRNA levels were normalized to *SDHA *(n = 3, mean and SD). There was no statistically significant difference between the curves for homozygous and wildtype LCLs at 6 hours or 8 hours.

In order to assess whether these variants modified the endogenous mRNA and protein levels we made use of LCLs established from blood samples from six breast cancer patients (BCLs), who had been genotyped previously for the three SNPs of interest and found to be either wild-type (three lines) or carriers of the variant alleles (three lines) (Hall and Vaslin, unpublished data). We examined the median mRNA levels in the two panels of BCLs and found a lower level in the homozygous compared to the wild-type lines although the result was not statistically significant (p = 0.49), with a wide interquartile range observed (Figure 2A). There was a borderline-significant reduction (p = 0.058) in median protein level in the homozygous carriers of the variant alleles compared to carriers of the wild-type alleles (Figure 2B and see Figure 2C for representative western blots). RNA stability was then assessed, by quantifying the transcript level at different times after the addition of Actinomycin D to the culture medium to arrest transcription, with mRNA quantified by quantitative RT-PCR (Figure 2D). Although mRNA decay was initially more rapid in the wild-type cells, mRNA levels appeared lower at 6 hours in homozygous mutant lines. However, the differences were not statistically significant (p = 0.21).

We then compared mRNA and protein levels with the genotype in bladder cancer patients whom we re-approached from our previous case-control study. Forty-nine hospital patients gave a blood sample (two samples in the case of one individual), and results were obtained for 46 individuals. Thirty samples were wild-type for all three variants (c.1496C > T, c.*611T > A and c.*618A > G), 16 were heterozygous for all three, and one was heterozygous for c.1496C > T but wild-type for c.*611T > A and c.*618A > G. There was no significant difference in median XPC protein expression or median mRNA level between lymphocytes from wild-type and heterozygous individuals (p = 0.12 and p = 0.29 respectively, Figure 2A and 2B).

## Discussion

The XPC SNP c.1496C > T (p.Ala499Val) is associated with increased bladder cancer risk [4,5]. However, c.1496C > T is not predicted to have functional effects by the analytical software tools SIFT (http://sift.jcvi.org/), Polyphen (http://genetics.bwh.harvard.edu/pph2/) and Pfam (http://pfam.sanger.ac.uk) and, compatible with the prediction software results, we recently found no effect of c.1496T on recruitment of XPC to sites of 408 nm laser microbeam damage [6]. However, individual SNPs can themselves be functional or alternatively in linkage disequilibrium with other variants which are causal [9] and we previously found the two 3'UTR *XPC *SNPs c.*611T > A and c.*618A > G to be in strong LD with c.1496C > T [4].

We therefore hypothesised that the two 3'UTR SNPs might have an effect on *XPC *mRNA stability and/or reduced mRNA transcription and/or translation, which could explain the association of c.1496T and increased bladder cancer risk. In this present study we demonstrated in a plasmid-based system that, in RT112M bladder cancer cell lines, the two 3'UTR SNPs are each associated with reduced protein and mRNA expression relative to wild type. These results were compatible with reduced mRNA stability/and or transcription. However, when we examined the impact of these variants on endogenous mRNA and protein levels in a small panel of LCLs homozygous for the wildtype or variant alleles whilst we found a borderline significant, approximately 30%, reduction in XPC protein expression, the mRNA expression result was more difficult to interpret as, although there was a lower mean mRNA level in homozygous carriers, there was a wide interquartile range and the result was not statistically significant.

Using mRNA stability assays there was also a suggestion of reduced stability in the LCL lines homozygous for the variant allele, intermediate between that of LCLs carrying the wildtype allele and XP-C GM15983 cells, which have undetectable mRNA and protein levels [10], although this was not statistically significant. Clearly larger panels of cell lines are needed and it is possible that cell type specific differences exist that means that LCLs are not an appropriate model system. The intrinsic stability of mRNA is determined by *cis*-acting sequences located within the 3'UTR as well as *trans*-acting RNA binding proteins [11]. 3'UTR SNPs may alter the binding of these proteins or alter the secondary structure of the 3'UTR [11-13].

In terms of our bladder cancer patients, unfortunately the terms of our ethical approval explicitly prevented us from directly contacting patients to enrich our population for known homozygous individuals. From our previous study we had predicted that four of our 46 re-approached patients would carry the homozygous genotype but, as there were no such individuals, we could not determine the effect of carriage of two variant alleles of the three SNPs in our patient samples. We could only establish that heterozygous carriage of the three variants had no influence on XPC protein or mRNA expression and so we were unable to determine whether our *in vitro *findings are replicated for endogenous XPC protein.

## Conclusions

Using an *in vitro *assay we found that the presence of either of the two *XPC *3'-UTR SNP variants, c.*611A and c.*618G, in strong LD with c.1496T, results in reduced mRNA stability and protein expression. As they are likely to either alter the structure of the 3'UTR or alter the binding of RNA binding proteins, it is perhaps not surprising that both exhibit similar phenotypes as they are only seven base pairs apart, and may be the causal variants that explain the associations previously found for c.1496T with increased bladder cancer risk.

## Abbreviations

ActD: actinomycin D; BCL: breast cancer lymphoblastoid cell lines; FBS: fetal bovine serum; LCLs: lymphoblastoid cell lines; LREC: Local Research Ethical Committee; MFI: mean fluorescent intensity; NER: nucleotide excision repair; SNPs: single nucleotide polymorphisms; UTR: untranslated region.

## Competing interests

The authors declare that they have no competing interests.

## Authors' contributions

BQ designed and performed the mRNA and protein assays and mRNA stability assays and wrote the first draft of the manuscript.

GBS designed and performed the 3'UTR plasmid reporter assays and wrote the relevant sections of the manuscript. FE checked the statistical analysis and reviewed the manuscript for statistical content. LV genotyped the breast cancer patient lymphoblastoid cell lines.

JB supervised the experimental work and advised on experimental design, and edited the manuscript. JH provided the breast cancer patient lymphoblastoid cell lines and redrafted and edited the manuscript. DTB reviewed and edited the manuscript. MAK provided RT112 cell lines and edited the manuscript. AEK designed the study, recruited the bladder cancer patients and drew their blood samples, supervised the experimental work, and redrafted the manuscript. All authors reviewed and approved the final manuscript.

## Pre-publication history

The pre-publication history for this paper can be accessed here:

http://www.biomedcentral.com/1471-2350/12/84/prepub
